# A screening of growth inhibitory activity of Iranian medicinal plants on prostate cancer cell lines

**DOI:** 10.1051/bmdcn/2018080208

**Published:** 2018-05-28

**Authors:** Majid Asadi-Samani, Mahmoud Rafieian-Kopaei, Zahra Lorigooini, Hedayatollah Shirzad

**Affiliations:** 1 Student Research Committee, Shahrekord University of Medical Sciences Shahrekord Iran; 2 Medical Plants Research Center, Basic Health Sciences Institute, Shahrekord University of Medical Sciences Shahrekord Iran; 3 Cellular and Molecular Research Center, Basic Health Sciences Institute, Shahrekord University of Medical Sciences Shahrekord Iran

**Keywords:** Prostate cancer, Phytochemical compounds, Drug discovery, Antiproliferation

## Abstract

Background: Prostate cancer has been known as one of the most common malignancy in the men and it is therefore very important to prevent and treat this cancer. In this study, the anticancer effects of 20 species of medicinal plants in Iran, especially those grown in Chaharmahal and Bakhtiari province, were investigated on prostate cancer cell lines to identify potential natural alternatives for the development of prostate cancer anticancer drugs.

Methods: The plants were gathered from Chaharmahal va Bakhtyari and their aerial parts extracted through maceration method using ethanol 70%. Anti-proliferative activity of extracts on PC-3, DU145 and HDF cell lines was evaluated by MTT assay 48 hours after treatment.

Results: *Euphorbia szovitsii Fisch. & C.A.Mey. and Achillea wilhelmsii* had anti-proliferative activity more than other plants on PC-3. Also IC50s for *Urtica dioica, Euphorbia szovitsii Fisch. & C.A.Mey.* and *Medicago sativa* were lower amount among the examined plants on Du-145.

Conclusion: According to our result, *Euphorbia szovitsii Fisch. & C.A.Mey., U. dioica* and *Medicago sativa* with good anti-proliferative activity can serve as an effective source of natural products to develop new antiprostate cancer drugs.

## 1. Introduction

Prostate cancer has been reported with a high incidence of 7.9%, representing the fourth leading cancer in the general population, according to the International Agency for Research on Cancer (IARC) of the World Health Organization released (WHO) in 2014 [1]. In a more recent report, 26% of newly diagnosed cases of cancer in the United States and 9% of men’s deaths are related to prostate cancer [2]. In other reports, the incidence rates of prostate cancer are different in different Asian countries, ranging from low incidence (2/100,000 population in Iran) to high incidence (20/100,000 population in the Philippines). In Iran, prostate cancer is one of the most common cancers among men, with the highest prevalence in Tehran (41.2%) and comparatively lower prevalence in other large and industrial provinces (36.8%), and small towns and villages (22.1%) [3-8]. These reports suggest that prostate cancer is one of the most common causes of mortality in men, comprising an important health issue, which makes its treatment essential. However, despite the many treatments for prostate cancer, due to drug resistance, several complications of used medications and treatments, the available treatment options have not been able to reduce the survival rates of prostate cancer patients and the survival rates remain far less-than-optimal [9-12].

Given the treatments that are currently being used to fight prostate cancer and associated complications, drug resistance especially in metastatic prostate cancers, ever-increasing costs of common treatments, and the increasing incidence of prostate cancer in both developing and developed countries, it is necessary to discover newer therapeutic approaches with higher efficacy to reduce the incidence and mortality of prostate cancer. In this regard, it is necessary to find cytotoxic plants against various cancers, especially prostate cancer, which, despite lower side effects, can replace chemotherapy and difficult treatments, and also be used for treatment-resistant cases [13-17]. These plants have high levels of phytochemicals that will have many therapeutic effects [18, 19]. Iran especially Chaharmahal and Bakhtiari province is rich in medicinal plants and many medicinal plants are found only in these areas and are native to these regions and the climatic conditions of these areas have caused these plants to contain high concentrations of phytochemical compounds with various therapeutic effects.

Taken together, our aim was to investigate certain species of medicinal plants in Iran, especially those growing in Chaharmahal va Bakhtiari province in Iran, whose effects have not yet been studied on prostate cancer cell lines but they are used as anticancer agents according to public beliefs and Iranian traditional medicine books, or whose anti-inflammatory and antioxidant effects were confirmed in studies so that they may be used to produce more efficient and novel drugs to treat prostate cancer.

## 2. Material and methods

### 2.1. Plant material

The plants were collected locally in different points of Chaharmahal va Bakhtiari province in Iran in May-Sep 2015 and botanically authenticated by Dr. Shirmardi (Research Center for Agricultural & Natural Resources, Shahrekord, Iran) and Miss S. Khademian (Department of Pharmacognosy, Faculty of Pharmacy, Shiraz University of Medical Sciences). *Stachys inflate, Salvia multicaulis Vahl, Hertia angustifolia, Sophora alopecuroides, Haplophyllum perforatum, Moriera spinosa Boiss., Teucrium orientale* L. subsp. taylori. (Boiss.), *Achillea wilhelmsii, Urtica dioica, Plantago lanceolata, Euphorbia microsciadia Boiss., Medicago sativa, Satureja bachtiarica, Acanthophyllum glandulosum* Bung. ex Boiss, *Onosma sericeum, Parietaria judaica, Phlomis persica, Ziziphora clinopodioides, Echinophora platyloba D.C, and Euphorbia szovitsii Fisch. & C.A.Mey* included in this study.

### 2.2. Preparation of extracts

The herbal samples were cleaned, shade dried in, pulverized to powder in a mechanical grinder and macerated in ethanol (70%) at room temperature for 72 hours. In the next step, the hydro alcoholic extracts were concentrated by a rotary evaporator under reduced pressure. Samples were dissolved in DMSO % 0.1 (dimethyl sulfoxide, Sigma) [20-23]. Finally, extracts were diluted in RPMI 1640 at concentration of 5 mg*/ml.*

### 2.3. Cell lines and culture medium

The following cancer cell lines were used for this study: PC-3 and DU145 (prostate cancer cell lines) and HDF (Human Dermal Fibroblasts) as non-cancer cell line. Cells were obtained from National Cell Bank of Iran (Pasteur Institute, Tehran, Iran).

Cells were cultured in RPMI1640 (Roswell Park Memorial Institute medium 1640; Gibco) with 1% penicillin-streptomycin and 10% FBS (Sigma) in a humidified atmosphere with 5% Co2 at 37°C throughout the assay.

### 2.4. Antiproliferative assay

3-[4, 5-dimethylthiazol-2-yl]-2,5 diphenyl tetrazolium bromide assay (MTT assay) was used for evaluating cell viability. The cells (PC-3, DU145, and HDF) cells were seeded in 96-well plates and incubated at 37°C. After 24 h of incubation, when cells reached more than 80-90% confluence, the medium was removed and the cells were treated with fresh medium containing various concentrations of plant extracts to be tested (10 μg/ml-1 mg/ml). After 48 h, the supernatant liquids were eliminated and a medium including MTT solution (0.5 mg*/ml)* was added to the wells which were incubated for 4 h. In the next step, supplements were eliminated, and the formazan crystals were dissolved in DMSO. The absorbance of the plates were determined at 570 nm with a reference wavelength of 630 nm in an enzyme linked immunosorbent assay (ELISA) reader.

The percentage of inhibition was measured as [1- (optical density of test/ optical density of negative control)] × 100. The IC50 value (the concentration with 50% cell inhibition) was calculated *via* the graph of inhibition percentage versus different extract concentrations.

### 2.5. Statistical analysis

The dose-response curves of the plants were fitted by means of the computer program GraphPad Prism 6.0 (GraphPad Software, USA), and IC50 was defined by regression analysis.

## 3. Results

In this study, 20 species of Iranian medicinal plants were investigated. The most of the plants were from Lamiaceae family (Table 1).

**Table 1:** Screened medicinal plants in this study.

No.	Scientific names	Persian name	Family	Herbarium code1
1	*Salvia multicaulis Vahl*	Gol arvaneh	Lamiaceae	Skums-301
2	*Stachys inflate*	Sonbole badkonaki	Lamiaceae	Skums-260
3	*Teucrium orientale* L. subsp. taylori. (Boiss.)	Maryam nokhodi sharghi Shirazi	Lamiaceae	Skums-522
4	*Satureja bachtiarica*	Marzeh Bakhtiyari	Lamiaceae	Skums-208
5	*Phlomis persica*	Goshbareh Irani	Lamiaceae	Skums-700
6	*Ziziphora clinopodioides*	Kakoti kohi	Lamiaceae	Skums-253
7	*Hertia angustifolia*	Karghich	Asteraceae	Skums-701
8	*Achillea wilhelmsii*	Bomadaran	Asteraceae	Skums-207
9	*Euphorbia microsciadia Boiss.*	Farfion	Euphorbiaceae	Skums-659
10	*Euphorbia szovitsii Fisch. & C.A.Mey.*	Farfion	Euphorbiaceae	Skums-935
11	*Sophora alopecuroides*	Talkhbayan	Fabaceae	Skums-258
12	*Medicago sativa*	Yonjeh	Fabaceae	Skums-742
13	*Haplophyllum perforatum*	Morde kazeb	Rutaceae	Skums-150
14	*Moriera spinosa Boiss.*	Kharmarjan	Brassicaceae	Skums-623
15	*Urtica dioica*	Gazaneh	Urticaceae	Skums-303
16	*Plantago lanceolata*	Kardi (Barhang sarneyzei)	Plantaginaceae	Skums-252
17	*Acanthophyllum glandulosum* Bung. ex Boiss	Chobak nekaei	Caryophyllaceae	Skums-896
18	*Onosma sericeum*	Gavzaban	Boraginaceae	Skums-841
19	*Parietaria judaica*	Goshmosh	Urticaceae	Skums-617
20	*Echinophora platyloba D.C*	Khosharozeh	Apiaceae	Skums-249

Anticancer activity (IC50) of the 20 medicinal plants on DU-145 and PC-3 has been shown in tables 2. Extracts with IC50>300 μg*/ml* in MTT assay were considered inactive.

**Fig.1:**
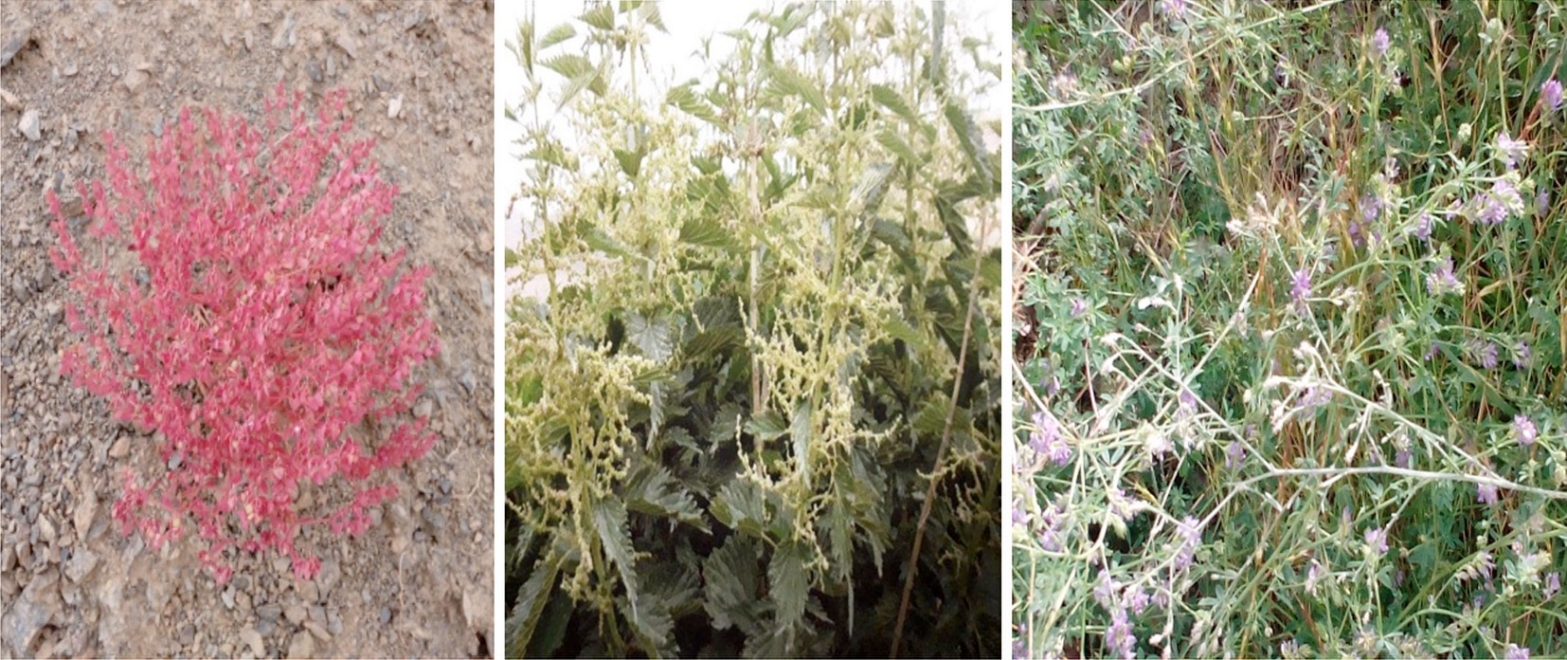
Pictures of more effective plants.

*Euphorbia szovitsii Fisch. & C.A.Mey.* (Fig. 1a) had the best anticancer effect on PC-3 compared other investigated medicinal plants. Also IC50s for *Urtica dioica* (Fig. 1b), *Euphorbia szovitsii Fisch. & C.A.Mey.* and *Medicago sativa* (Fig. 1c) were lower amount among the examined plants on Du-145.

For investigating the cytotoxicity effect of medicinal plants gathered from Chaharmahal va Bakhtiari province on normal cells we investigated the effect of ethanolic extract of effective medicinal plants on HDF cell line (Table 3). In IC50 concentration, *Urtica dioica.* and *Euphorbia szovitsii Fisch. & C.A.Mey.* don’t have any effects on HDF cells.

**Table 2:** Anticancer effect of the ethanolic extract of medicinal plants gathered from Chaharmahal and Bakhtiari province on prostate cancer cell lines.

	PC-3			Du-145		
Scientific names	IC50 (μg/m/)	95% Confidence Intervals	R	IC50 (μg/m/)	95% Confidence Intervals	R
*Echinophora platyloba* D.C	300<	-	-	300<	-	-
*Medicago sativa*	300<	-	-	77	55-107	0.8952
*Sophora alopecuroides*	300<	-	-	192	182-202	0.9983
*Onosma sericeum*	300<	-	-	300<	-	-
*Haplophyllum perforatum*	266	158-449	0.7676	300<	-	-
*Moriera spinosa* Boiss.	300<	-	-	300<	-	-
*Euphorbia microsciadia Boiss.*	222	189-248	0.9884	300<	-	-
*Hertia angustifolia*	300<	-	-	216	188-249	0.9991
*Acanthophyllum glandulosum* Bung. ex Boiss	300<	-	-	300<	-	-
*Salvia multicaulis* Vahl	240	197-294	0.9468	143	116-179	0.9570
*Phlomis persica*	300<	-	-	300<	-	-
*Euphorbia szovitsii Fisch. & C.A.Mey.*	111	62-127	0.973856	56	43-73	0.9285
*Achillea wilhelmsii*	193	115-262	0.9221	151	136-170	0.9960
*Urtica dioica*	300<	-	-	37	34-41	0.9863
*Teucrium orientale L. subsp. taylori. (Boiss.)*	300<	-	-	300<	-	-
*Plantago lanceolata*	300<	-	-	300<	-	-
*Stachys inflata*	300<	-	-	300<	-	-
*Ziziphora clinopodioides*	300<	-	-	300<	-	-
*Satureja bachtiarica*	300<	-	-	300<	-	-
*Parietaria judaica*	300<	-	-	300<	-	-

IC50 determined by MTT colorimetric assay.

**Table 3:** Cell viability of the ethanolic extract of effective medicinal plants gathered from Chaharmahalva Bakhtiari province on HDF.

Scientific names	IC50 (μg/*ml*)	95% Confidence Intervals	R
*Medicago sativa*	448	382-526	0.9676
*Sophora alopecuroides*	1236	647-2360	0.8279
*Euphorbia microsciadia Boiss.*	451	336-605	0.9463
*Acanthophyllum glandulosum Bung. ex Boiss*	74	70-78	0.9964
*Euphorbia szovitsii Fisch. & C.A.Mey.*	412	313-542	0.9870
*Urtica dioica*	1222	1020-1450	0.9540
*Salvia multicaulis Vahl*	1350	875-1500	0.8450
*Achillea wilhelmsii*	1120	745-1200	0.9452
*Hertia angustifolia*	375	250-456	0.9336

## 4. Discussion

According to the results of studies and also the report of IARC of the WHO in 2014 [6], despite extensive studies on the discovery of anticancer drugs, the incidence and prevalence of various cancers remain high and in many cases, drug resistance leads to lack of appropriate response; therefore, it is essential to conduct further studies and discover drugs with more potent effects that can serve as alternatives to chemical drugs and reduce the side effects of current medications and therapies. Phytotherapy is one of the approaches that have been used to treat various cancers in recent years [13-17]. In this regard, the present study was carried out to screen medicinal plant species in Iran, especially Chaharmahal and Bakhtiari province, on prostate cancer cell lines. Of the studied 20 plant species, *Euphorbia szovitsii* fisch & C.A.Mey and *Achillea wilhelmsii* exerted relatively higher anticancer effects on the PC-3 cell line and *Urtica dioica, Euphorbia szovitsii* Fisch. & C.A.Mey and *Medicago sativa* exerted comparatively more potent anticancer effects on the DU-145 cell line.

So far, the preventive and anticancer effects of many medicinal plants, as well as the effects of their derivatives on different cell lines, have been studied. In a review (2015), the effects of 34 Iranian medicinal plants tested for their anticancer effects on different cell lines and with animals and human subjects were reported [23]. *U. dioica,* which has been investigated for its effect on prostate cancer cell lines including LNCaP and hPCPs, is one of the plants in Iran whose effects on some prostate cancer cell lines have been studied [24]. In our study, the effect of this plant on the other two cell lines of prostate cancer, i.e. PC-3 and DU-145, was investigated. A study (2014) showed that the cytotoxic effect of aqueous *U. dioica* extract on LNCaP cell line is mediated by apoptosis and oxidative stress [25]. Aqueous *U. dioica* extract results in significant inhibition of adenosine deaminase (ADA) activity in prostate tissue [26].

*Medicago sativa,* as one of the other plants in our study, has good anti-proliferative activity on DU-145 cell line. In the previous study, anticancer effect of *M. sativa* on multidrug-resistant tumor cells lines has been reported. *M. sativa* could induce apoptosis in these cells lines [27]. Also some of isolated compounds from *M. sativa* have been indicated to have antitumor activity against different cancer cell lines such as leukemia, cervix and breast cancer [28, 29]. The results of present study and other studies that have examined the effect of *U. dioica* and *M. sativa* on cancers suggest them as effective medicinal plants in the prevention and treatment of cancers.

*Thymus vulgaris, Taverniera spartea, Camellia sinensis, Ferula gummosa, Allium sativum, Curcuma longa and Zingiber officinale* are other species in Iran, which are similar to the studied plants studied in the current work in terms of plant families or chemical compounds, and their anticancer effects on prostate cancer have also been investigated [30-35]. However, according to the available results, none of the plant species in Iran have yet been studied for their effects on prostate cancer cell lines (DU-145 and PC-3).

In the present study, *Euphorbia szovitsii* Fisch. & C.A.Mey, that is a species of the Euphorbia genus, was found to have better antitumoural effects on both prostate cancer cell lines than other plants under study. The plants from the Euphorbia genus have traditionally been used to treat inflammation and tumors [36, 37]. Besides that, cytotoxic and immunosuppressive effects have been reported for the hydroalcoholic extracts of other species of the Euphorbia genus. In addition, in recent studies, the anticancer effects of other plants of the Euphorbia genus and also their compounds, have been reported specifically on various cancer cell lines, suggesting that the plants of this genus have an acceptable anti-cancer potential [38-45]. The results of our study are in line with these studies and *Euphorbia szovitsii* Fisch. & C.A.Mey has an acceptable potential for anticancer effects on prostate cancer.

## 5. Conclusion

According to our results, *E. szovitsii* Fisch. & C.A.Mey. with good anti-proliferative activity on both prostate cancer cell lines and *U. dioica* and *M. sativa* with good anti-proliferative activity on DU-145 cell line can serve as an effective source of natural products to develop new anti-prostate cancer drugs.

## Conflicts of interest statement

The authors declare no conflict of interest.
